# Hepatitis E Virus Transmission from Wild Boar Meat

**DOI:** 10.3201/eid1112.051041

**Published:** 2005-12

**Authors:** Tian-Cheng Li, Katsumi Chijiwa, Nobuyuki Sera, Tetsuya Ishibashi, Yoshiki Etoh, Yuji Shinohara, Yasuo Kurata, Miki Ishida, Shigeru Sakamoto, Naokazu Takeda, Tatsuo Miyamura

**Affiliations:** * National Institute of Infectious Diseases, Tokyo, Japan; † Fukuoka Institute of Health and Environmental Sciences, Fukuoka, Japan; ‡ Tagawa Health, Welfare, and Environment Office, Fukuoka, Japan; § Fukuoka Prefectual Government, Fukuoka, Japan; ¶ Iizuka Hospital, Fukuoka, Japan

**Keywords:** Hepatitis E, hepatitis E virus, zoonosis, wild boar, foodborne infection, dispatch

## Abstract

We investigated a case of hepatitis E acquired after persons ate wild boar meat. Genotype 3 hepatitis E virus (HEV) RNA was detected in both patient serum and wild boar meat. These findings provided direct evidence of zoonotic foodborne transmission of HEV from a wild boar to a human.

Hepatitis E virus (HEV), a causative agent of human hepatitis E, is a single-stranded positive-sense RNA virus recently classified as the sole member of the genus *Hepevirus* in the family *Hepeviridae* (*1**,**2*). HEV is transmitted primarily by the fecal-oral route through contaminated drinking water. However, recent studies have demonstrated that various animal species have serum antibodies to HEV, suggesting that hepatitis E is a zoonotic disease (*3*). In Japan, 4 hepatitis E cases have been linked directly to eating raw deer meat (*4*), and several cases of acute hepatitis E have been epidemiologically linked to eating undercooked pork liver or wild boar meat (*5**,**6*). These cases provide convincing evidence of zoonotic food-borne HEV transmission. We report direct evidence of HEV transmission from a wild boar to a human.

## The Study

A 57-year-old woman came to Iizuka Hospital on March 12, 2005, with malaise and anorexia. Although she was a healthy hepatitis B virus carrier and negative for serologic markers of hepatitis A and C, testing upon admission showed elevated levels of liver enzymes (alanine aminotranferase 752 IU/L, aspartate aminotransferase 507 IU/L, and γ-glutamyl transpeptidase 225U/L). A serum sample collected on March 16 was positive for both immunoglobulin M (IgM) and IgG antibodies to HEV when tested by an antibody enzyme-linked immunosorbent assay using recombinant viruslike particles (*7*). This led to the diagnosis of hepatitis E. The hepatitis was typical, acute, and self-limiting, and the patient recovered by the end of March.

The patient's husband traditionally hunted boar for food 3 or 4 times a year, and she had eaten boar meat on 2 occasions. With her husband, she ate the meat as part of a hot pot on December 28, 2004, 11 weeks before her illness, and again, grilled, on January 19, 2005, along with 10 other people (including her husband) 8 weeks before her illness. Disease did not develop in the other 10 people. Except for this wild boar meat, the patient had not eaten meat or liver from other wild animals. Since she had not traveled abroad in the past 30 years, transmission must have occurred in Japan. Two portions of meat from the wild boar (meats 1 and 2) eaten on December 28, 2004, and 1 portion from the other wild boar (meat 3) eaten on January 19, 2005, remained and were frozen.

Juice was obtained from the sliced meat by centrifugation at 10,000 × *g* for 15 min. The supernatant was used for RNA extraction. A nested reverse transcription–polymerase chain reaction (RT-PCR) was conducted to amplify part of open reading frame 2 (ORF2), which corresponds to nucleotides (nt) 5939–6297 of the genotype 1 HEV genome (GenBank D10330), with external sense primer HEV-F1 (5´-TAYCGHAAYCAAGGHTGGCG-3´) and antisense primer HEV-R2 (5´-TGYTGGTTRTCRTARTCCTG-3´). A nested PCR was conducted with internal sense primer HEV-F2 (5´-GGBGTBGCNGAGGAGGAGGC-3´) and internal antisense primer HEV-R1 (5´-CGACGAAATYAATTCTGTCG-3´). This procedure allows amplification of HEV 1, 3, and 4 genotypes. A PCR product of 359 bp including the primer sequences was obtained from meat 3 by nested PCR. However, meats 1 and 2 were negative. HEV RNA was not detected in the patient's serum by the same amplification method. This may have resulted from an extremely small amount of RNA.

New primers for the nested RT-PCR were designed for a region within the 359 base region based on the meat 3 sequences, which corresponded to nt 5983–6243. The first PCR was performed with external sense primer HEV-WB-F1 (5´-ACCTCTGGCCTGGTAATGCT-3´) and antisense primer HEV-WB-R2 (5´-GAGAAGCGTATCAGCAAGGT-3´). The nested PCR was performed with internal sense primer HEV-WB-F2 (5´-TATTCATGGCTCTCCTGTCA-3´) and internal antisense primer HEV-WB-R1 (5´-ACAGTGTCAGAGTAATGCCT-3´). These primers allowed amplification of 281 nt, including the primer sequences from the patient serum collected on March 16, 2005. In contrast, meats 1 and 2 were negative with these new primers.

To further analyze the RNA in the patient serum and meat 3, RNA genomes encoding an entire ORF2 were amplified as overlapping segments, nucleotide sequences were determined, and phylogenetic analysis was carried out with avian HEV as an outgroup. Avian HEV is a causative agent of chicken hepatitis-splenomegaly syndrome (*8*). Two sequences, 1 from the patient (DQ079629) and the other from meat 3 (DQ079630), were classified into genotype 3 (Figure). Only 1 nt difference was observed in the 1,980 nt of the entire ORF2; the nucleotide sequence identity was 99.95%. The difference was not accompanied by any amino acid changes. These data demonstrated that HEV infection was transmitted from the wild boar meat to the patient on January 19, 2005.

**Figure:**
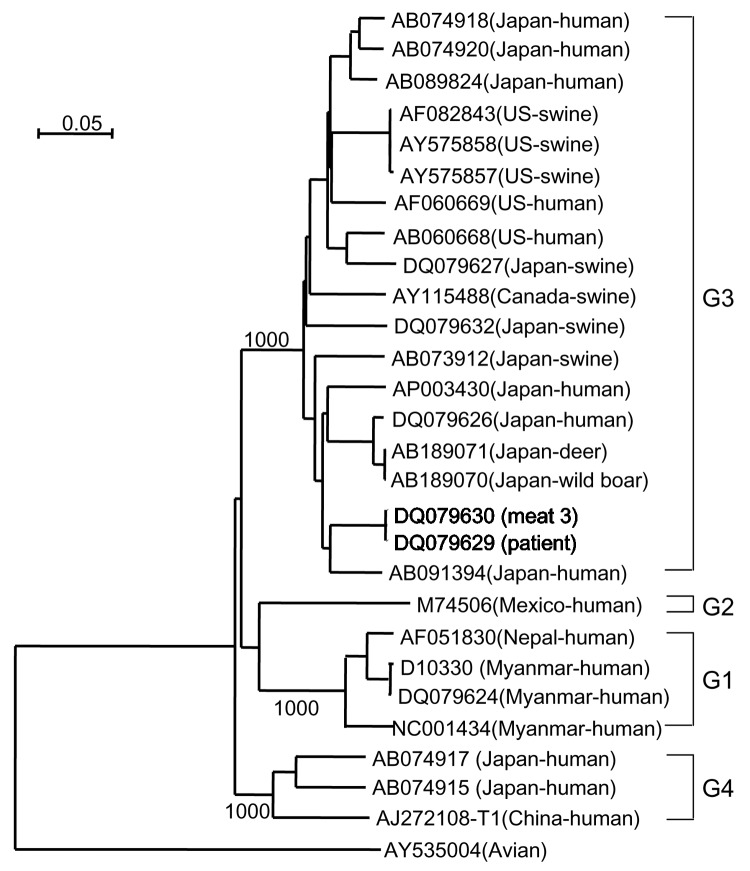
Phylogenetic tree of hepatitis E virus (HEV) reconstructed with avian HEV as an outgroup. Nucleotide sequences of the entire open reading frame 2 were analyzed by the neighbor-joining method. The bootstrap values correspond to 1,000 replications. The 2 nucleotide sequences characterized in this study are shown in bold. The horizontal scale bar at the top left indicates nucleotide substitutions per site.

## Conclusions

Currently, deer, pig, and wild boar are suspected sources of foodborne zoonotic transmission of HEV in Japan, and genotypes 3 and 4 of HEV are believed to be indigenous (*4**–**6**,**9**,**10*). Direct evidence for transmission of genotype 3 HEV from animals to humans was observed in acute hepatitis in 4 persons who had eaten uncooked deer meat that contained ≈10^7^ copies of HEV RNA (*4*). However, the rare finding of HEV antibody-positive deer in Japan suggest that deer are not the major zoonotic reservoir of HEV in this country (*11*). In contrast, high antibody-positive rates in domestic pig and wild boar, including HEV genotypes 3 and 4, have been frequently detected, suggesting that persons who eat uncooked meat are at risk for infection with HEV (*12**,**13*). This report is the first to provide direct evidence of zoonotic foodborne genotype 3 HEV transmission from wild boar to a human.
